# Cost analysis in a Traditional, Complementary and Integrative Medicine unit in Brazil

**DOI:** 10.11606/s1518-8787.2020054001649

**Published:** 2020-11-27

**Authors:** Marcone César Tabosa Assunção, Camilla Maria Ferreira de Aquino, Islândia Maria Carvalho de Sousa, Manoel Raymundo de Carvalho, Vitor Pereira Jordão, Adriana Falangola Benjamin Bezerra

**Affiliations:** I Universidade Federal de Pernambuco Hospital das Clínicas RecifePE Brasil Universidade Federal de Pernambuco. Hospital das Clínicas. Recife, PE, Brasil; II Instituto Federal de Pernambuco Departamento de Enfermagem Abreu e LimaPE Brasil Instituto Federal de Pernambuco, Campus Abreu e Lima. Departamento de Enfermagem. Abreu e Lima, PE, Brasil; III Fundação Oswaldo Cruz Instituto Aggeu Magalhães Departamento de Saúde Coletiva RecifePE Brasil Fundação Oswaldo Cruz. Instituto Aggeu Magalhães. Departamento de Saúde Coletiva. Recife, PE, Brasil; IV Universidade Federal de Pernambuco Centro de Ciências da Saúde Departamento de Gerontologia RecifePE Brasil Universidade Federal de Pernambuco. Centro de Ciências da Saúde. Departamento de Gerontologia. Recife, PE, Brasil; V Alegre centro de análise do comportamento RecifePE Brasil Alegre centro de análise do comportamento. Direção Clínica. Recife, PE, Brasil; VI Universidade Federal de Pernambuco Centro de Ciências da Saúde Departamento de Medicina Social RecifePE Brasil Universidade Federal de Pernambuco. Centro de Ciências da Saúde. Departamento de Medicina Social. Recife, PE, Brasil

**Keywords:** Integrative Medicine, economics, Complementary Therapies, economics, Health Services Administration, economics, Health Economics

## Abstract

**OBJECTIVE:**

To analyze the costs of a specialized service in Traditional Complementary and Integrative Medicines (TCIM) in Northeast Brazil to provide data on the cost linked to the implementation and maintenance of services of this nature and to identify the average cost per user for the Unified Health System.

**METHODS:**

This is a partial, descriptive, quantitative economic assessment, which used secondary data, later grouped in Microsoft Excel spreadsheets. The method used to analyze such costs was absorption costing, from which the service was divided into three costing centers: productive, administrative and auxiliary.

**RESULTS:**

After analyzing the data, the total cost of the service in 2014 was estimated at R$ 1,270,015.70, with a proportion of 79.69% of direct costs. The average cost per user in this period was R$ 36.79, considering the total of 34,521 users in individual and collective practices.

**CONCLUSIONS:**

The service has a cost per user compatible with a specialized service; however, TCIM offers a comprehensive and holistic approach, which can have a positive impact on quality of life.

## INTRODUCTION

The increasing insertion of Traditional, Complementary and Integrative Medicines (TCIM)^1^ in various health systems in the world has broadened the discussion about their costs and the possibility of integration. The social, institutional and academic recognition of MTCI reinforces the idea that the biomedical model coexists with other forms of care, in a cultural context characterized by pluralistic therapy^2,3^.

TCIM focuses on the integral approach of the human being, bringing together the physical, psychological, cultural and social systems^4^. In Brazil, they are called in the Unified Health System (SUS) of Integrative and Complementary Practices in Health (ICPH)^1^, coming from different rationalities in health. The supply of ICPH in SUS has recently expanded, totaling 29 practices from complex systems or therapeutic resources, being: homeopathy, phytotherapy/medicinal plants, acupuncture/traditional Chinese medicine, anthroposophical medicine, social thermalism/crenotherapy, art therapy, ayurveda, biodance, circular dance, meditation, music therapy, naturopathy, osteopathy, chiropractic, reflexotherapy, reiki, shantala, integrative community therapy, yoga, aromatherapy, apitherapy, bioenergetics, family constellation, chromotherapy, geotherapy, hypnotherapy, laying on of hands, ozone therapy and flower therapy^5^.

Such expansion of supply deserves more in-depth analyses and studies in the field of economic evaluation that support decision-making in SUS on this topic.

This reality provides subsidies for the emergence of new services within SUS that respond to the wishes of users, health professionals and managers, as well as the scientific community. However, TCIM are weakly institutionalized and distributed heterogeneously in the country^1^. This occurs due to several factors, such as: absence of trained professionals, lack or insufficiency of financial resources to implement and maintain the services and low knowledge about the costs and effectiveness of these services^6^.

Public administration has shown a trend towards more professional management, with cost assessment. This view of managerial efficiency has been implemented in public health institutions^7^. Given its limits, this assessment can subsidize investment in the quality of care as it allows reallocating and optimizing resources. Regarding TCIM services, this parallel with cost accounting is poorly documented, which generates administrative distortions and difficulties in estimating the value of the service and/or TCMI procedures for SUS. Considering the lack of scientific production that focuses on TCIM costs, our study aimed to estimate the costs of a specialized TCIM service in Northeast Brazil.

## METHODS

It is a cost analysis^8^ from the perspective of public management, using absorption costing from a public unit specialized in TCIM, offering differentiated individual and collective practices and the production of therapeutic supplies, located in João Pessoa (Paraíba). The absorption costing method aims to identify costs as direct, since it groups them into centers that depend on them to carry out their core activities. Only items that cannot be exclusively allocated to the respective cost centers will be classified as indirect costs and, subsequently, apportioned among all cost centers^9^.

The cost was estimated in relation to the user and the service. Regarding the user, the estimate was made by dividing the total cost (direct and indirect) by the total number of users served in the year. Regarding the service, the estimate considered individual and collective practices. In the first ones, the service of a user by a therapist is considered, that is, a 1:1 ratio. In the second ones, each therapeutic group was estimated at 10 users per service, with 1 therapist for 10 users. This estimate was made according to the direct observation of the researchers and through the reports of the professionals of the unit, which was built exclusively to offer TCIM, meeting the specificities of these practices.

The individual practices include: acupuncture; auriculotherapy; homeopathy; phytotherapy; art therapy; floral therapy; chiropractic; reiki; aromatherapy; massage therapy; Ayurvedic therapy and massage; foot reflexology and naturology, which also includes iridology and geotherapy. As for the collective ones, they are summarized in: biodance; circular dances; Tai Chi Chuan; community therapy; yoga; taking care of the caregiver; preventive self-massage; art therapy; healing through self-expression (plastic arts); meditation; corporal conscience; family constellation; dance of the sacred feminine and circle of pregnant women.

Data were collected between 2015 and 2016, referring to 2014, as it is the last complete year at the beginning of the research. Data were obtained through the analysis of documents and other electronic cost records, in addition to direct observation of the physical area of the service, resources, activities developed and interviews with professionals and managers of the unit (Chart).

The data collected were grouped into three costing centers: productive, administrative and auxiliary. This definition was based on the purpose of each sector and its relationship with services produced and offered to SUS users.

The productive costing center consisted of 8 offices, a compounding pharmacy, 5 rooms for collective practices, a toy library and the auditorium. The administrative costing center consisted of a warehouse, reception room, administration room, board and meeting room. The auxiliary costing center was defined by sectors that served as support for the operational operation: 2 break rooms, bathrooms, Medical and Statistical Archive Service (MSAS), service area, kitchen, pantry, storage of cleaning material.

The reception/security service was not allocated in the cost centers, being identified as an indirect cost. The utility room, the internal garden and building expenses also fell into this category.

TCIM services are characterized by the low use of equipment with high technological density. In this sense, professionals have an important share of costs. At the unit, 47 employees are responsible for the operation of the service, 31 of which are allocated to the productive costing center, 8 to the administrative and 4 to the auxiliary. The reception/security service is represented by three professionals. The greatest concentration of the employees is in the productive sector, with emphasis on holistic therapists, with 11 representatives, who offer individual and collective assistance in various specialties.

**Box:** Identification of expense items, their source and their respective estimation methods in 2014.

Type of Data	Data Source	Estimation
Break room, office and hygiene supplies	Input output report. Municipal Health Secretariat of João Pessoa (Paraíba)	Sum of the value of items used in the period and distribution according to the costing center.
Inputs for final activities	Input output report. Municipal Health Secretariat of João Pessoa (Paraíba)	Sum of the value of all items used during customer service and allocation in the productive costing center.
Human Resources	Management Annual Report (MAR). Municipal Health Secretariat of João Pessoa (Paraíba)	Sum of all professionals’ salaries with respective social charges (INSS, FGTS, vacation, thirteenth salary) and distribution according to the purpose within the service.
Investment materials (machinery, furniture and equipment)	Municipal Health Secretariat of João Pessoa (Paraíba)	The purchase value was used to estimate the depreciation of investment materials, in order to identify its cost in the study period. The costs of the materials were then distributed according to their purpose in the service.
Equipment for structuring the public compounding pharmacy	Specialized Care Management. Municipal Health Secretariat of João Pessoa (Paraíba)	The purchase value was used to estimate the depreciation of investment materials, in order to identify its cost in the study period. This set of equipment is used for end-of-service activities.
Building data	Board of the TCIM Health Unit. Municipal Health Secretariat of João Pessoa (Paraíba)	The value of the property and its depreciation was estimated to find its cost in the study period. In addition, other maintenance costs were estimated. All estimated costs were apportioned uniformly between the costing centers.
Electricity, water and sewage	Board of the TCIM Health Unit. Municipal Health Secretariat of João Pessoa (Paraíba)	All water/sewage and electricity bills were added during the study period, and the amount was evenly distributed among the costing centers.
Telephony	Central Telephone Coordination. Municipal Health Secretariat of João Pessoa (Paraíba)	All accounts for the study period were added up, and the amounts estimated were allocated to the administrative costing center.
Service quantitative	Management Annual Report (MAR). Municipal Health Secretariat. João Pessoa (Paraíba)	Sum of all services reported in the BAP and subsequent classification in individual or collective.

Source: Authors.

BPA: Bulletin of Ambulatory Procedures.

This study is part of the project *Apuração dos custos das medicinas/práticas integrativas e complementares na saúde mental em Pernambuco* (ApuraSMPIC – Determination of the costs of medicines/integrative and complementary practices in mental health in Pernambuco), Called MTCI/CNPq/MS-SCTIE-Decit no. 07/2013, approved by the CAAE Ethics Committee: 07270212.4. 0000.5208.

## RESULTS

In the assessment of the production in 2014, there was an increase of 48.82% in the opening of medical records in relation to the previous year. Collective service also grew by 102.96%, totaling 26,479 users in the year. In individual practices, there was a reduction of 184 appointments compared with 2013, with 8,042, totaling 34,521 users treated in the year.

Among individual practices, acupuncture was the most used, with 3,122 visits, followed by flower therapy, with 1,114, and chiropractic, with 638.

When considering unit costs, with their due depreciation, the unit cost amount was estimated at R$ 1,270,015.70 in 2014. According to the classification adopted in this study, direct costs were responsible for R$ 1,012,140.45 (79.7%) of the total expenses of the health unit; and indirect costs, for R$ 257,875.24 (20.3%). Direct costs were subdivided into: human resources – (R$ 934,173.33; 92.30%), representing the salaries of professionals, added to all labor obligations (thirteenth salary, FGTS, vacation, INSS); depreciation of machinery, equipment and furniture (R$ 13,797.22; 1.36%) and consumables (R$ 64,169.90; 6.34%) (Table 1).

**Table 1:** Cost categories, with annual value of the indirect and direct costs of the cost centers (in reais) and percentage representation over the total cost of the unit specialized in TCIM in 2014.

	Human Resources	Furniture, machinery and equipment (depreciation)	Consumables	Building costs (depreciation, water and electricity)	Subtotal (R$)
Direct cost of administrative costing center	166,379.00	2,451.12	11,428.42	-	180,258.54 (14.19%)
Direct cost of the auxiliary costing center	86,881.12	1,280.15	5,967.78	-	94,129.05 (7.41%)
Direct cost of the productive costing center	680,913.21	10,065.95	46,773.70	-	737,752.86 (58.09%)
Indirect Costs	31,520.00	711.93	1.896,00	223,747.32	257,875.24 (20,30%)

Total costs	965,693.33	14,509.15	66,065.90	223,747.32	1,270,015.70 (100%)

Of the estimated consumption material, the largest portion refers to break room, cleaning and hygiene supplies. The inputs used within the productive costing center had less contribution. The provision of services in the unit is characterized by the user/therapist relationship, without the need for many inputs besides essential oils, acupuncture needles, essences, procedure gloves and medicinal plants.

Indirect costs totaled R$ 257,875.25. Of this amount, 86.76% (R$ 223,747.32) comprise building costs, in which the greatest impact is related to the depreciation of the property. The building had an estimated value of R$ 2,738,190.00; considering the depreciation rate for this type of investment at 4%^10^, the cost of the property was R$ 109,527.60 in the period studied. Building costs are also represented by expenses with water/sewage and electricity, estimated at R$ 114,221.76 in 2014. Expenses related to the Urban Property Tax (IPTU) and garbage collection are also part of the building costs; however, this study disregarded them, since the unit is exempt from these taxes for being a public institution. The professional activities that are part of the indirect costs are related to the safety of the health unit. Expenses related to these workers comprise 12.22% (R$ 31,520.00) of indirect costs (Table 1) and relate to wages and all labor obligations.

The unit’s total cost for providing services was R$ 1,270,015.70 in 2014, with a monthly average of R$ 105,834.64. Collective practices accounted for 76.70% of the total attendances and, consequently, individual practices accounted for 23.30%.

The distribution of direct costs by costing center was made based on the allocation of human resources in the respective centers, since personnel expenses represented the greatest impact in this category. The productive costing center gathers most professionals (31), which is why it had a greater impact on the unit’s costs (57.75%) (Table 1).

The criterion chosen to apportion the indirect costs between the costing centers was the number of rooms (spaces) in the TCIM service, considering that the indirect costs are related to the acquisition and maintenance of the building infrastructure, such as property depreciation, expenses with water supply and electricity. The service in question has 29 spaces, including offices, auditoriums, bathrooms, meeting room, among others. As for the distribution of spaces, the productive costing center is responsible for the largest number of rooms, with 14 of them, followed by the auxiliary center, with 10, and the administrative center, with 5. Considering this, 48.27% of the unit’s indirect costs were allocated to the productive costing center (Table 2).

**Table 2:** Direct cost and respective apportionments of indirect costs by costing center (in reais) of the unit specialized in TCIM in 2014.

Costing center	Direct cost (R$)	Apportionment of indirect costs (R$)	Total cost of the costing center (R$)
Administrative costing center	180,259.12	44,457.69 (17.24%)	224,716.69
Costing center auxiliary	94,129.06	88,915.38 (34.48%)	183,044.12
Costing center productive	737,752.87	124,476.37 (48.27%)	862,229.24

The distinction between individual and collective practices was considered to estimate the cost of care in this service. The average cost of user service was R$ 36.79. This allowed us to identify that the service in collective practices represents about 13% of the estimated average value per user of individual practices (Table 3).

**Table 3:** Cost of service per user of individual and collective practices in the specialized unit, considering 10 users per collective practice.

Service Types	Quantity of appointments	Costs by category (R$)	Service costs (R$)
Individual Practices	8,042	888,629.98	110.49
Collective Practices	26,470	381,385.71	14.40

Total	34,512	1,270,015.70	36.79 (Mean Cost)

## DISCUSSION

The unit analyzed was structured exclusively to offer TCIM and, thus, portrayed all the costs involved in individual and collective care in TCIM in SUS. The planning and implementation process for this service was carried out by the municipal management, despite the existence of a gap in Brazilian municipalities regarding public policies on financing, implementation and sustainability of these units and their role in the Health Care Network^11^. The few services with these characteristics have a “provisional” character and low institutionality^12^, which directly interferes with their financing. The Ministry of Health estimate is that 25% of Brazilian municipalities have services that offer TCIM, mainly inserted in Primary Health Care (PHC) (78%), specifically in the Family Health Strategy (FHS), and only 18% are insert into specialized care^13^. Despite this scenario, no studies that specifically addressed the costs of TCIM in SUS were found, a fact that may discourage managers from implementing such services in the public network.

The most relevant costs for the operation of the service were employees’ salaries and their respective employer’s charges, reflecting the reality of most health services^16^. This characteristic can be strategic, as it is effective without being directly connected to the use of high technologies and, therefore, has more potential to reduce costs. The training of professionals in the field of TCIM is an important gap for the development of other health rationales in SUS. Despite this reality, it is essential that studies on the effectiveness of TCIM address the costs of inputs and their economic results^17^.

The unit value of R$ 36.79 per service is considered compatible with biomedical services of medium complexity; however, the service provided in TCIM is also characterized by being resolutive and does not generate extra demands, such as specialized exams and other invasive procedures, in addition to those performed during the service. Despite this, this field is also part of a scenario marked by lack of resources and unlimited demand, requiring sustainable financing and management mechanisms. With a similar methodology (absorption costing), the cost of an outpatient appointment was estimated at R$ 36.24, in a state maternity hospital in Santa Catarina. The costs are compatible with those of the service at the TCIM unit, even considering peculiarities inherent to both studies, such as complexity of equipment, supplies, apportionment criteria and purpose of services^16^.

The cost-effectiveness of TCIM is documented only in the international literature. A Dutch study showed that users had fewer hospitalizations and prescription drugs and lower expenses with general practitioners who perform homeopathy, acupuncture and anthroposophy^18^. The Chinese traditional medicine has been cost-effective for health systems and insurances^19^. We found that acupuncture reduces referrals to physical therapy and rheumatology in the primary health care services where it was introduced^22^.

To establish stages of opening, maintenance and dimensioning health units specialized in TCIM, it is essential to analyze the costs in order to identify the investment necessary to offer these services. Cost management is part of the efficient management of any entity that offers services or products, whether in the private or public sphere^23^.

The service is characterized by low technological density, with raw material that requires little expenditure of financial resources. The data were verified with the analysis of the investment and consumption materials of the productive costing center, in which the greatest financial impact is caused by human resources.

The result of the cost assessment needs to consider the integral and holistic approach adopted in the TCIM. Studies on cost-effectiveness can contribute to expand and contextualize the characteristics of health care in this field. Economic assessments are recommended, such as cost-effectiveness, which aims to assess the relationship between what is spent and the effectiveness of TCIM for SUS users.

The consolidation of innovative policies, such as the TCIM offer, is directly influenced by the disclosure of the indications and effectiveness of these practices. The aim is to increase the number of users and professionals who know its benefits, either through direct access or through professional referral to services of this nature. Initiatives like these can dilute the cost per user of TCIM, since units aimed at such services have a high fixed cost.

The organizational structure experienced in some countries, such as the USA, which host TCIM and biomedical services in the same physical space, can be a strategy in Brazil according to each local context, given the lack of resources in many municipalities to implement an exclusive unit^24^. In fact, this has occurred in the provision of TCIM in PHC, but it needs a back-up network to receive due support. According to the study by Sousa & Tesser^11^, the type 3 insertion model (PHC with matrix support) is one of the potential ways of expanding MTCI in SUS. In the national and international literature, the discussion is scarce about the costs of implantation and maintenance of health units specialized in TCIM, given they have financing through outpatient production. Recent research found three exclusive units in TCIM in Brazil, two in Recife (Pernambuco) and another in João Pessoa (Paraíba)^1^. These are important cases for professionals and managers to understand aspects of their implementation and financial sustainability.

At the time of the study, different individual and collective practices offered were not part of the scope of institutionalized care in SUS, which changed with the publication of two ordinances, which may alter the registered production. But since financing has not been guaranteed, this fact is unlikely to change revenue collection. Therefore, TCIM continues in Brazil without specific investment or inducing resources.

The arbitrariness during the appropriation and apportionment of indirect costs is another factor that makes it difficult to compare studies, being common in cost management. This happens, most of the time, due to the lack of knowledge of managers or the lack of apportionment parameters, especially when it comes to services that provide different services^9^. A study carried out with private hospitals in João Pessoa (Paraíba) found that only 10% of the sample’s managers shared all the indirect costs^25^.

This fact is ratified by this study, which has as a limitation the impossibility of accurately apportioning indirect costs, due to the lack of available data. Another limitation is the estimate of 10 users per group of collective practice, since this number was estimated empirically, not statistically.

TCIM is an emerging and complex field, with varying acceptance within society and the scientific community. Although public policies and studies subsidize their use in a formal way, the implementation of these practices remains irregular and little disseminated in SUS, with important gaps to be filled in the health economy. This study can support further deepening and analysis in this regard.

## References

[1] 1. Sousa IMC, Bodstein, RCA, Tesser CD, Santos FAS, Hortale VA. Práticas integrativas e complementares: oferta e produção de atendimentos no SUS e em municípios selecionados. Cad Saude Publica. 2012;28(11):2143-54. 10.1590/S0102-311X2012001100014 23147956

[2] 2. World Health Organization. Traditional medicine strategy: 2014-2023. Geneva: WHO; 2014.

[3] 3. Andrade JT, Costa LFA. Medicina complementar no SUS: práticas integrativas sob a luz da Antropologia médica. Saude Soc. 2010;19(3):497-508. 10.1590/S0104-12902010000300003

[4] 4. Melo SCC, Santana RG, Santos DC, Alvim NAT. Práticas complementares de saúde e os desafios de sua aplicabilidade no hospital: visão de enfermeiros. Rev Bras Enfermagem. 2013;66(6):840-6. 10.1590/S0034-71672013000600005 24488454

[5] 5. Ministério da Saúde (BR. Portaria Nº 702, de 21 de março de 2018. Incluídas no Sistema Único de Saúde novas práticas na Política Nacional de Práticas Integrativas e Complementares (PNPIC). Diário Oficial da União. 22 mar 2018; Seção 1:65.

[6] 6. Bonacim CAG, Araújo AMP. Gestão de custos aplicada a hospitais universitários públicos: a experiência do Hospital das Clínicas da Faculdade de Ribeirão Preto da USP. Rev Adm Publica. 2010;44(4):903-31. 10.1590/S0034-76122010000400007

[7] 7. Cogan S. Activity-basedCosting (abc): a poderosa estratégia empresarial. São Paulo: Pioneira; 1994.

[8] 8. Ugá MAD. Instrumentos de avaliação econômica dos serviços de saúde: alcances e limitações. In: Piola SF, Vianna SM, organizadores. Economia da saúde. 3.ed. Brasília, DF: IPEA; 2002. Cap 9, p. 209-27.

[9] 9. Ministério da Saúde; Organização Mundial da Saúde. Introdução à gestão de custos em saúde. Brasília, DF: Editora do Ministério da Saúde; 2013. (Série Gestão e Economia em Saúde; vol 2).

[10] 10. Secretaria da Receita Federal (BR). Instrução normativa SRF Nº 162, de 31 de dezembro de 1998. Fixa o prazo de vida útil e taxa de depreciação dos bens que relaciona. Diário Oficial da União. 7 jan 1999 [cited 2016 Mar 18]; Seção 1:5. Available from: http://normas.receita.fazenda.gov.br/sijut2consulta/link.action?visao=anotado&idato=15004

[11] 11. Sousa IMC, Tesser, CD. Medicina Tradicional e Complementar no Brasil: inserção no Sistema Único de Saúde e integração com a atenção primária. Cad Saude Publica. 2017;33(1):e00150215. 10.1590/0102-311x00150215 28125126

[12] 12. Nascimento MC, Barros NF, Nogueira MI, Luz MT. A categoria racionalidade médica e uma nova epistemologia em saúde. Cienc Saude Coletiva. 2013;18(12):3595-604. 10.1590/S1413-81232013001200016 24263876

[13] 13. Ministério da Saúde (BR). Práticas integrativas e complementares crescem na rede SUS de todo o Brasil. Brasília, DF; atual jun 2018 [cited 2018 Jul 16]. Available from: http://dab.saude.gov.br/portaldab/noticias.php?conteudo=_&cod=2205

[14] 14. Ministério da Saúde (BR). Práticas integrativas e complementares em saúde: uma realidade no SUS. Rev Bras Saude Fam. 2008 [cited 2015 Feb 2]. Available from: http://189.28.128.100/dab/docs/publicacoes/revistas/revista_saude_familia18_especial.pdf

[15] 15. Ministério da Saúde (BR),. Secretaria de Atenção à Saúde, Departamento de Atenção Básica. Manual de implantação de serviços de práticas integrativas e complementares no SUS. Brasília, DF; 2018.

[16] 16. Raupp FM, Crispim CH, Almeida ES. Gestão de custos hospitalares por meio do custeio por absorção: o caso da Maternidade Carmela Dutra. Rev Inform Contab. 2007;1(2):120-33.

[17] 17. Herman PM, Poindexter BL, Witt CM, Eisenberg DM. Are complementary therapies and integrative care cost-effective? A systematic review of economic evaluations. BMJ Open. 2012;2(5):e001046. 10.1136/bmjopen-2012-001046 PMC3437424 22945962

[18] 18. Kooreman P, Baars EW. Patients whose GP knows complementary medicine tend to have lower costs and live longer. Eur J Health Econ. 2012;13(6):769-76. 10.1007/s10198-011-0330-2 PMC3482459 21695547

[19] 19. Baars EW, Kooreman P. A 6-year comparative economic evaluation of healthcare costs and mortality rates of Dutch patients from conventional and CAM GPs. BMJ Open 2014;4(8):e005332. 10.1136/bmjopen-2014-005332 PMC4156802 25164536

[20] 20. Herman PM, Poindexter BL, Witt CM, Eisenberg DM. Are complementary therapies and integrative care cost-effective? A systematic review of economic evaluations. BMJ Open. 2012;2(5):e001046. 10.1136/bmjopen-2012-001046 PMC3437424 22945962

[21] 21. Lorenc A, Feder G, MacPherson H, Little P, Mercer SW, Sharp D. Scoping review of systematic reviews of complementary medicine for musculoskeletal and mental health conditions. BMJ Open. 2018;8(10):e020222. 10.1136/bmjopen-2017-020222 PMC6196876 30327397

[22] 22. Ross J. An audit of the impact of introducing microacupuncture into primary care. Acupunct Med 2001;19(1):43–5. 10.1136/aim.19.1.43 11471583

[23] 23. Martins E. Contabilidade de custos. 10. ed. São Paulo: Atlas; 2010.

[24] 24. Griffin KH, Nate KC, Rivard RL, Christianson JB, Dusek JA. Referrals to integrative medicine in a tertiary hospital: findings from electronic health record data and qualitative interviews. BMJ Open. 2016;6(7):e012006. 10.1136/bmjopen-2016-012006 PMC4964262 27456330

[25] 25. Lucena WGL, Brito LASN. Um estudo do tratamento dos custos indiretos nos hospitais privados de João Pessoa-PB. Qualitas. 2010;9(2):33-48. 10.18391/qualitas.v9i2.569

