# Supplementary material for: Cost analysis in a Traditional, Complementary and Integrative Medicine unit in Brazil
Source: Rev Saude Publica. 2020 Nov 27;54:145. doi: 10.11606/s1518-8787.2020054001649 (PMC7702377; doi:10.11606/s1518-8787.2020054001649)
Supplement: Translation

# Translation: Análise de custos em unidade de Medicina Tradicional, Complementar e Integrativa no Brasil

**Authors:** Marcone César Tabosa Assunção, Camilla Maria Ferreira de Aquino, Islândia Maria Carvalho de Sousa, Manoel Raymundo de Carvalho, Vitor Pereira Jordão, Adriana Falangola Benjamin Bezerra

**Affiliations:** I Brasil Universidade Federal de Pernambuco. Hospital das Clínicas. Recife, PE, Brasil; II Brasil Instituto Federal de Pernambuco, Campus Abreu e Lima. Departamento de Enfermagem. Abreu e Lima, PE, Brasil; III Brasil Fundação Oswaldo Cruz. Instituto Aggeu Magalhães. Departamento de Saúde Coletiva. Recife, PE, Brasil; IV Brasil Universidade Federal de Pernambuco. Centro de Ciências da Saúde. Departamento de Gerontologia. Recife, PE, Brasil; V Brasil Alegre centro de análise do comportamento. Direção Clínica. Recife, PE, Brasil; VI Brasil Universidade Federal de Pernambuco. Centro de Ciências da Saúde. Departamento de Medicina Social. Recife, PE, Brasil

**Keywords:** Medicina Integrativa, economia, Terapias Complementares, economia, Administração de Serviços de Saúde, economia, Economia da Saúde

## Abstract

**OBJETIVO:**

Analisar os custos de um serviço especializado em Medicinas Tradicionais Complementares e Integrativas (MTCI) no Nordeste brasileiro, com o intuito de fornecer dados sobre o custo atrelado à implantação e manutenção de serviços dessa natureza e identificar o custo médio por usuário para o Sistema Único de Saúde.

**MÉTODOS:**

Trata-se de uma avaliação econômica do tipo parcial, com caráter descritivo, de natureza quantitativa, que utilizou dados secundários, posteriormente agrupados em planilhas do *Microsoft Excel*. O método utilizado para analisar tais custos foi o de custeio por absorção, a partir do qual o serviço foi dividido em três centros de custeio: produtivo, administrativo e auxiliar.

**RESULTADOS:**

Após a análise dos dados, o custo total do serviço em 2014 foi estimado em R$ 1.270.015,70, com proporção de 79,69% de custos diretos. O custo médio por usuário neste período foi R$ 36,79, considerando o total de 34.521 usuários em práticas individuais e coletivas.

**CONCLUSÕES:**

O serviço apresenta um custo por usuário compatível com um serviço especializado, contudo, as MTCI oferecem abordagem compreensiva e holística, as quais podem impactar de forma positiva a qualidade de vida.

## INTRODUÇÃO

A crescente inserção das Medicinas Tradicionais, Complementares e Integrativas (MTCI)^1^ em vários sistemas de saúde do mundo tem ampliado a discussão acerca dos seus custos e possibilidade de integração. O reconhecimento social, institucional e acadêmico das MTCI reforça a ideia de que o modelo biomédico convive com outras formas de cuidado, em um contexto cultural caracterizado pelo pluralismo terapêutico^2,3^.

As MTCI enfocam a abordagem integral do ser humano, reunindo os sistemas físico, psicológico, cultural e social^4^. No Brasil, são chamadas no Sistema Único de Saúde (SUS) de Práticas Integrativas e Complementares em Saúde (PICS)^1^, provenientes de diferentes racionalidades em saúde. A oferta das PICS no SUS tem se ampliado recentemente, totalizando 29 práticas provenientes de sistemas complexos ou recursos terapêuticos, sendo: homeopatia, fitoterapia/plantas medicinais, acupuntura/medicina tradicional chinesa, medicina antroposófica, termalismo social/crenoterapia, arteterapia, ayurveda, biodança, dança circular, meditação, musicoterapia, naturopatia, osteopatia, quiropraxia, reflexoterapia, reiki, shantala, terapia comunitária integrativa, yoga, aromaterapia, apiterapia, bioenergética, constelação familiar, cromoterapia, geoterapia, hipnoterapia, imposição de mãos, ozonioterapia e terapia de florais^5^.

Tal ampliação de oferta merece análises mais aprofundadas e estudos no campo da avaliação econômica que subsidiem a tomada de decisão no SUS acerca deste tema.

Essa realidade proporciona subsídios para o surgimento de novos serviços dentro do SUS que respondam aos anseios dos usuários, dos profissionais e gestores de saúde, bem como da comunidade científica. No entanto, as MTCI são fragilmente institucionalizadas e distribuídas de forma heterogênea no país^1^. Isso ocorre devido a vários fatores, tais como: ausência de profissionais capacitados, falta ou insuficiência de recursos financeiros indutores para implantar e manter os serviços e baixo conhecimento acerca dos custos e da efetividade desses serviços^6^.

A administração pública tem demonstrado tendência para uma gestão mais profissionalizada, com avaliação de custos. Essa visão de eficiência gerencial tem sido implantada nas instituições públicas de saúde^7^. Dados os seus limites, essa avaliação pode subsidiar o investimento na qualidade da assistência à medida que permite realocar e otimizar recursos. No que se refere aos serviços de MTCI, esse paralelo com a contabilidade de custos é pouco documentado, o que gera distorções administrativas e dificuldade de estimar o valor do serviço e/ou procedimentos de MTCI para o SUS. Considerando o cenário de ausência de produção científica que enfoque os custos em MTCI, o estudo que gerou este manuscrito teve por objetivo estimar os custos de um serviço especializado em MTCI no Nordeste brasileiro.

## MÉTODOS

Trata-se de uma análise de custos^8^ a partir da perspectiva da gestão pública, utilizando o custeio por absorção de uma unidade pública especializada em MTCI, com oferta de práticas individuais e coletivas diferenciadas e produção de insumos terapêuticos, localizada em João Pessoa (PB). O método de custeio por abrsorção visa identificar os custos como diretos, uma vez que os agrupa em centros que dependem destes para realizar suas atividades-fim. Apenas itens que não podem ser exclusivamente alocados nos respectivos centros de custo serão classificados como custos indiretos e, posteriormente, rateados entre todos os centros de custo^9^.

O custo foi calculado em relação ao usuário e ao atendimento. No que tange ao usuário, o cálculo foi feito dividindo o custo total (direto e indireto) pelo total de usuários atendidos no ano. Em relação ao atendimento, o cálculo considerou práticas individuais e coletivas. Nas primeiras, considera-se o atendimento de um usuário por terapeuta, ou seja, a relação é de 1 para 1. Nas segundas, cada grupo terapêutico foi estimado em 10 usuários por atendimento, sendo 1 terapeuta para 10 usuários. Essa estimativa foi realizada de acordo com a observação direta dos pesquisadores e por meio dos relatos dos profissionais da unidade, a qual foi construída exclusivamente para oferecer MTCI, atendendo às especificidades dessas práticas.

Dentre as práticas individuais ofertadas estão: acupuntura; auriculoterapia; homeopatia; fitoterapia; arteterapia; terapia floral; quiropraxia; reiki; aromaterapia; massoterapia; terapia e massagem ayurvédica; reflexologia podal e naturologia, que também inclui iridologia e geoterapia. Quanto às coletivas, resumem-se em: biodança; danças circulares; tai chi chuan; terapia comunitária; yoga; cuidando do cuidador; automassagem preventiva; arteterapia; cura em expressão (artes plásticas); meditação; consciência corporal; constelação familiar; dança do sagrado feminino e círculo de gestantes.

A coleta de dados foi realizada entre 2015 e 2016, referente ao ano de 2014, por se tratar do último ano completo no início da pesquisa. Os dados foram obtidos por meio da análise de documentos e outros registros eletrônicos de custos, além da observação direta da área física do serviço, dos recursos, das atividades desenvolvidas e entrevista com os profissionais e gestores da unidade (Quadro).

**Quadro:** Identificação dos itens de despesas, sua fonte e seus respectivos métodos de cálculo em 2014.

Tipo de Dado	Fonte dos Dados	Cálculo
Insumos de copa, escritório e produtos de higiene	Relatório de saída de insumos. Secretaria Municipal de Saúde de João Pessoa (PB)	Soma do valor dos itens utilizados no período e distribuição de acordo com o centro de custeio.
Insumos para atividades fins	Relatório de saída de insumos. Secretaria Municipal de Saúde João Pessoa (PB)	Soma do valor de todos os itens utilizados durante o atendimento ao usuário e alocação no centro de custeio produtivo.
Recursos Humanos	Relatório Anual de Gestão (RAG). Secretaria Municipal de Saúde João Pessoa (PB)	Soma de todos os salários dos profissionais com os respectivos encargos sociais (INSS, FGTS, férias, décimo terceiro) e distribuição de acordo com a finalidade dentro do serviço.
Materiais de investimento (máquinas, mobílias e equipamentos)	Secretaria Municipal de Saúde de João Pessoa (PB)	O valor de compra foi utilizado para calcular a depreciação dos materiais de investimento, a fim de identificar seu custo no período estudado. Posteriormente os custos dos materiais foram distribuídos de acordo com sua finalidade no serviço.
Equipamentos para estruturação da farmácia com manipulação pública	Gerência de Atenção Especializada. Secretaria Municipal de Saúde de João Pessoa (PB)	O valor de compra foi utilizado para calcular a depreciação dos materiais de investimento, a fim de identificar o seu custo no período estudado. Esse conjunto de equipamentos é utilizado para atividades-fim do serviço.
Dados prediais	Direção da Unidade de Saúde em MTCI. Secretaria Municipal de Saúde de João Pessoa (PB)	Foi estimado o valor do imóvel e, posteriormente, calculada a sua depreciação para estimar seu custo no período estudado. Além disso, calcularam-se as outras despesas com sua manutenção. Todos os custos estimados foram rateados uniformemente entre os centros de custeio.
Energia elétrica, água e esgoto	Direção da Unidade de Saúde em MTCI. Secretaria Municipal de Saúde de João Pessoa (PB)	Somaram-se todas as contas de água/esgoto e energia elétrica durante o período estudado, e o valor foi rateado uniformemente entre os centros de custeio.
Telefonia	Coordenação Central Telefônica. Secretaria Municipal de Saúde de João Pessoa (PB)	Somaram-se todas as contas do período estudado, e os valores apurados foram alocados ao centro de custeio administrativo.
Quantitativo de atendimento	Relatório Anual de Gestão (RAG). Secretaria Municipal de Saúde. João Pessoa (PB)	Soma de todos os atendimentos informados no BPA e posterior classificação em individual ou coletivo.

Fonte: Elaborado pelos autores.

BPA: Boletim de Procedimentos Ambulatoriais.

Os dados coletados foram agrupados em três centros de custeio: produtivo, administrativo e auxiliar. Essa definição teve por base a finalidade de cada setor e sua relação com serviços produzidos e oferecidos aos usuários do SUS.

O centro de custeio produtivo era composto por 8 consultórios, farmácia de manipulação, 5 salas de práticas coletivas, brinquedoteca e o auditório. O centro de custeio administrativo era constituído por almoxarifado, sala de recepção, sala de administração, diretoria e sala de reuniões. O centro de custeio auxiliar foi definido por setores que serviam como suporte para o funcionamento operacional: 2 copas, banheiros, Serviço de Arquivo Médico e Estatístico (Same), área de serviço, cozinha, despensa, depósito de material de limpeza.

O serviço de portaria/segurança não foi alocado nos centros de custos, sendo identificado como custo indireto. A sala de utilidade, o jardim interno e as despesas prediais também se enquadraram nessa categoria.

Os serviços de MTCI têm como característica o baixo uso de equipamentos com alta densidade tecnológica. Nesse sentido, os profissionais têm importante participação nos custos. Na unidade, 47 funcionários são responsáveis pelo funcionamento do serviço, sendo 31 alocados no centro de custeio produtivo, 8 no administrativo e 4 no auxiliar. O serviço de portaria/segurança é representado por 3 profissionais. A maior concentração dos funcionários da unidade está no setor produtivo, com destaque para os terapeutas holísticos, com 11 representantes, que oferecem atendimentos individuais e coletivos em diversas especialidades.

Este estudo faz parte do projeto “Apuração dos custos das medicinas/práticas integrativas e complementares na saúde mental em Pernambuco – ApuraSMPIC”, Chamada MTCI/CNPq/MS-SCTIE-Decit nº 07/2013, aprovado no Comitê de Ética CAAE: 07270212.4.0000.5208.

## RESULTADOS

Na avaliação da produção no ano de 2014, observou-se acréscimo de 48,82% na abertura de prontuários em relação ao ano anterior. Os atendimentos coletivos também cresceram, em 102,96%, totalizando 26.479 usuários no ano. Nas práticas individuais, houve redução de 184 atendimentos em relação a 2013, com 8.042, totalizando 34.521 usuários atendidos no ano.

Dentre as práticas individuais, a acupuntura foi a mais utilizada, com 3.122 atendimentos, seguida pela terapia floral, com 1.114, e pela quiropraxia, com 638.

Ao considerar os custos unitários, com suas devidas depreciações, o montante dos custos da unidade foi estimado em R$ 1.270.015,70 no ano de 2014. De acordo com a classificação adotada neste estudo, os custos diretos foram responsáveis por R$ 1.012.140,45 (79,7%) do total de despesas da unidade de saúde; e os indiretos, por R$ 257.875,24 (20,3%). Os custos diretos foram subdivididos em: recursos humanos – (R$ 934.173,33;92,30%), representando os salários dos profissionais, somados a todas as obrigações trabalhistas (décimo terceiro, FGTS, férias, INSS); depreciação de máquinas, equipamentos e móveis (R$ 13.797,22; 1,36%) e materiais de consumo (R$ 64.169,90; 6,34%) (Tabela 1).

**Tabela 1:** Categorias de custos, com valor anual dos custos indiretos e diretos dos centros de custo (em reais) e representatividade percentual sobre o custo total da unidade especializada em MTCI em 2014.

	Recursos Humanos	Móveis, máquinas e equipamentos (depreciação)	Material de consumo	Custos prediais (depreciação, água e luz)	Subtotal (R$)
Custo direto do centro de custeio administrativo	166.379,00	2.451,12	11.428,42	-	180.258,54 (14,19%)
Custo direto do centro de custeio auxiliar	86.881,12	1.280,15	5.967,78	-	94.129,05 (7,41%)
Custo direto do centro de custeio produtivo	680.913,21	10.065,95	46.773,70	-	737.752,86 (58,09%)
Custos indiretos	31.520,00	711,93	1.896,00	223.747,32	257.875,24 (20,30%)

Total de custos	965.693,33	14.509,15	66.065,90	223.747,32	1.270.015,70 (100%)

Do material de consumo estimado, a maior parcela é referente a insumos da copa, limpeza e higiene. Os insumos utilizados dentro do centro de custeio produtivo tiveram contribuição menor. A prestação de serviços na unidade é caracterizada pela relação usuário/terapeuta, sem a necessidade de muitos insumos além de óleos essenciais, agulhas de acupuntura, essências, luvas de procedimento e plantas medicinais.

Os custos indiretos totalizaram R$ 257.875,25. Desse montante, 86,76% (R$ 223.747,32) compreendem os custos prediais, nos quais o maior impacto está relacionado à depreciação do imóvel. O prédio teve o valor estimado em R$ 2.738.190,00; levando em consideração a taxa de depreciação para esse tipo de investimento em 4%^10^, o custo do imóvel foi de R$ 109.527,60 no período estudado. Os custos prediais também são representados por despesas com água/esgoto e energia elétrica, estimadas em R$ 114.221,76 em 2014. Despesas relacionadas ao Imposto Predial e Territorial Urbano (IPTU) e à coleta de lixo também fazem parte dos custos prediais, entretanto, esse estudo os desconsiderou, visto que a unidade é isenta desses impostos por se tratar de uma instituição pública. As atividades profissionais que fazem parte dos custos indiretos estão relacionadas à segurança da unidade de saúde. As despesas referentes a esses trabalhadores englobam 12,22% (R$ 31.520,00) dos custos indiretos (Tabela 1) e dizem respeito aos salários e a todas as obrigações trabalhistas.

O custo total de prestação de serviços da unidade foi de R$ 1.270.015,70 no ano de 2014, com média mensal de R$ 105.834,64. As práticas coletivas representaram 76,70% do total de atendimentos e, consequentemente, as práticas individuais representaram 23,30%.

A distribuição dos custos diretos por centro de custeio foi realizada a partir da alocação dos recursos humanos nos respectivos centros, visto que os gastos com pessoal representaram o maior impacto nessa categoria. Constatou-se que o centro de custeio produtivo reúne a maior parte dos profissionais (31), por isso teve maior impacto nos custos da unidade (57,75%) (Tabela 1).

O critério escolhido para ratear os custos indiretos entre os centros de custeio foi a quantidade de salas (espaços) no serviço de MTCI, tendo em vista que os custos indiretos estão relacionados à aquisição e manutenção da infraestrutura predial, tais como depreciação do imóvel, gastos com abastecimento hídrico e energia elétrica. O serviço em questão possui 29 espaços, incluindo consultórios, auditórios, banheiros, sala de reunião, entre outros. Quanto à distribuição de espaços, o centro de custeio produtivo é responsável pela maior quantidade de salas, com 14 delas, seguido pelo centro auxiliar, com 10, e pelo administrativo com 5. Diante disto, 48,27% dos custos indiretos da unidade foram alocados ao centro de custeio produtivo (Tabela 2).

**Tabela 2:** Custo direto e respectivos rateios dos custos indiretos por centro de custeio (em reais) da unidade especializada em MTCI em 2014.

Centro de custeio	Custo direto (R$)	Rateio dos custos indiretos (R$)	Custo total do centro de custeio (R$)
Centro de custeio administrativo	180.259,12	44.457,69 (17,24%)	224.716,69
Centro de custeio auxiliar	94.129,06	88.915,38 (34,48%)	183.044,12
Centro de custeio produtivo	737.752,87	124.476,37 (48,27%)	862.229,24

Considerou-se a distinção entre práticas individuais e coletivas para estimar o custo do atendimento neste serviço. O custo médio do atendimento ao usuário foi de R$ 36,79. Isso permitiu identificar que o atendimento em práticas coletivas representa cerca de 13% do valor médio estimado por usuário de prática individual (Tabela 3).

**Tabela 3:** Custo do atendimento por usuário de práticas individuais e coletivas na unidade especializada, considerando 10 usuários por prática coletiva.

Tipos de Atendimento	Quantidade de atendimentos	Custos por categoria (R$)	Custos por atendimento (R$)
Práticas Individuais	8.042	888.629,98	110,49
Práticas Coletivas	26.470	381.385,71	14,40

Total	34.512	1.270.015,70	36,79 (Custo médio)

## DISCUSSÃO

A unidade analisada estruturou-se exclusivamente para ofertar MTCI e, desta forma, retratou todos os custos envolvidos no atendimento individual e coletivo em MTCI no SUS. O processo de planejamento e implantação desse serviço foi executado pela gestão municipal, apesar de existir uma lacuna nos municípios brasileiros quanto às políticas públicas que versam sobre financiamento, implantação e sustentabilidade dessas unidades e a sua função na Rede de Atenção à Saúde^11^. Os poucos serviços com estas características possuem caráter “provisório” e de baixa institucionalidade^12^, o que interfere diretamente no seu financiamento. A estimativa do Ministério da Saúde é que 25% dos municípios brasileiros possuem serviços que ofertam MTCI, principalmente inseridos na Atenção Primária à Saúde (APS) (78%), especificamente na Estratégia de Saúde da Família (ESF), e apenas 18% se inserem na atenção especializada^13^. Mesmo diante desse cenário, não foram encontrados estudos que tratassem especificamente dos custos das MTCI no SUS no Brasil, fato que pode desencorajar gestores a implantar tais atendimentos na rede pública.

Os custos mais relevantes para o funcionamento do serviço foram com salários dos funcionários e seus respectivos encargos patronais, refletindo a realidade da maioria dos serviços de saúde^16^. Essa característica pode ser estratégica, pois produz efetividade sem estar diretamente conectada com o uso de altas tecnologias e, portanto, tem mais potencial de reduzir custos. A formação de profissionais no campo da MTCI constitui uma importante lacuna para o desenvolvimento de outras racionalidades em saúde no SUS. Apesar dessa realidade, é essencial que os estudos que versam sobre a efetividade da MTCI tratem dos custos dos insumos e dos seus resultados econômicos^17^.

O valor unitário de R$ 36,79 por atendimento é considerado compatível com serviços biomédicos de média complexidade, entretanto, o atendimento efetuado em MTCI também é caracterizado por ser resolutivo e não gerar demandas extras, como exames especializados e outros procedimentos invasivos, além dos realizados no ato do atendimento. Apesar disso, esse campo também faz parte de um cenário marcado por escassez de recursos e demanda ilimitada, necessitando de mecanismos sustentáveis de financiamento e gestão. Com metodologia semelhante (custeio por absorção), estimou-se o custo de uma consulta ambulatorial em R$ 36,24, em uma maternidade estadual de Santa Catarina. Os custos são compatíveis com os do atendimento na unidade de MTCI, mesmo considerando peculiaridades inerentes aos dois estudos, como complexidade de equipamentos, insumos, critérios de rateio e finalidade dos serviços^16^.

O custo-efetividade da MTCI é documentado apenas na literatura internacional. Um estudo holandês evidenciou que usuários tiveram menos internações e prescrição de drogas e menores despesas com médicos generalistas que realizam homeopatia, acupuntura e antroposófia^18^. A medicina tradicional chinesa tem sido custo-efetiva para os sistemas e seguros de saúde^19^. Constatou-se que a acupuntura diminui os encaminhamentos para fisioterapia e reumatologia nos serviços de atenção primária em saúde em que foi introduzida^22^.

Para estabelecer etapas de abertura, manutenção e dimensionamento de unidades de saúde especializadas em MTCI é imprescindível analisar os custos, a fim de identificar o investimento necessário para ofertar esses serviços. O gerenciamento dos custos faz parte da gestão eficiente de qualquer entidade que ofereça serviços ou produtos, seja no âmbito privado ou público^23^.

O serviço tem como característica a baixa densidade tecnológica, com matéria-prima que requer pouco dispêndio de recursos financeiros. Esse dado foi constatado com a análise dos materiais de investimento e de consumo do centro de custeio produtivo, no qual o impacto financeiro maior é causado pelos recursos humanos.

O resultado da avaliação do custo precisa considerar a abordagem integral e holística adotada nas MTCI. Estudos sobre custo-efetividade podem contribuir para ampliar e contextualizar as características do cuidado em saúde nesse campo. Recomendam-se avaliações econômicas como a de custo-efetividade, que tem o intuito de aferir a relação entre o que se gasta e a efetividade das MTCI para o usuário do SUS.

A consolidação de políticas inovadoras, como a oferta de MTCI, é influenciada diretamente pela divulgação das indicações e efetividade dessas práticas. O intuito é ampliar o número de usuários e profissionais que conhecem seus benefícios, seja pelo acesso direto ou por meio de encaminhamento profissional para serviços dessa natureza. Iniciativas como essas podem diluir o custo por usuário de MTCI, visto que unidades voltadas a tais atendimentos apresentam um custo fixo alto.

A estrutura organizacional vivenciada em alguns países, como os EUA, que abrigam os serviços de MTCI e biomédicos no mesmo espaço físico, pode ser uma estratégia no Brasil de acordo com cada contexto local, haja vista a falta de recursos em muitos municípios para implantar uma unidade exclusiva^24^_._ De fato, isso tem ocorrido na oferta de MTCI na APS, mas esta necessita de uma rede de retaguarda para receber o devido apoio. Conforme o estudo de Sousa e Tesser^11^, o modelo de inserção tipo 3 (APS com matriciamento) é uma das formas potenciais de expansão da MTCI no SUS. Na literatura nacional e internacional a discussão é escassa sobre os custos de implantação e manutenção de unidades de saúde especializadas em MTCI, haja vista o fato de terem financiamento via produção ambulatorial. Pesquisa recente encontrou três unidades exclusivas em MTCI no Brasil, duas em Recife (PE) e outra em João Pessoa (PB)^1^. Trata-se de casos importantes para profissionais e gestores compreenderem aspectos de sua implantação e sustentabilidade financeira.

Na época do estudo, diferentes práticas individuais e coletivas ofertadas não faziam parte do escopo de atendimentos institucionalizados no SUS, o que se modificou com a publicação de duas portarias, as quais poderão alterar a produção registrada. Mas como não foi garantido financiamento, este fato provavelmente não vai alterar a captação de receitas. Portanto, as MTCI seguem no Brasil sem investimento específico ou recurso indutor.

A arbitrariedade durante a apropriação e o rateio dos custos indiretos é outro fator que dificulta a comparação entre estudos, sendo recorrente na gestão de custos. Isso acontece, na maioria das vezes, por desconhecimento dos gestores ou por falta de parâmetros de rateio, principalmente quando se trata de serviços que prestam atendimentos distintos^9^. Estudo realizado com hospitais privados em João Pessoa (PB) verificou que apenas 10% dos gestores da amostra rateava a totalidade dos custos indiretos^25^.

Tal fato é ratificado por este estudo, que apresenta como limitação a impossibilidade de ratear de forma precisa os custos indiretos, devido à escassez de dados disponíveis. Outra limitação identificada refere-se à estimativa de 10 usuários por grupo de prática coletiva, uma vez que este número foi estimado de forma empírica, não estatística.

A MTCI constitui um campo emergente e complexo, com aceitação variada dentro da sociedade e da comunidade científica. Apesar de políticas públicas e estudos subsidiarem sua utilização de maneira formal, a implantação dessas práticas permanece irregular e pouca difundida no SUS, com importantes lacunas a serem preenchidas na economia da saúde. Acredita-se que este estudo pode subsidiar novos aprofundamentos e análises nesse sentido.
